# Duration Dependent Effect of Static Stretching on Quadriceps and Hamstring Muscle Force

**DOI:** 10.3390/sports6010024

**Published:** 2018-03-13

**Authors:** Leyla Alizadeh Ebadi, Ebru Çetin

**Affiliations:** Sports Sciences Faculty, Gazi University, 06560 Ankara, Turkey; ecetin@gazi.edu.tr

**Keywords:** static stretching, isokinetic strength, hamstring, quadriceps

## Abstract

The aim of this study was to determine the acute effect of static stretching on hamstring and quadriceps muscles’ isokinetic strength when applied for various durations to elite athletes, to investigate the effect of different static stretching durations on isokinetic strength, and finally to determine the optimal stretching duration. Fifteen elite male athletes from two different sport branches (10 football and five basketball) participated in this study. Experimental protocol was designed as 17 repetitive static stretching exercises for hamstring and quadriceps muscle groups according to the indicated experimental protocols; ((A) 5 min jogging; (B) 5 min jogging followed by 15 s static stretching; (C) 5 min jogging followed by 30 s static stretching; (D) 5 min jogging, followed by static stretching for 45 s). Immediately after each protocol, an isokinetic strength test consisting of five repetitions at 60°/s speed and 20 repetitions at 180°/s speed was recorded for the right leg by the Isomed 2000 device. Friedman variance analysis test was employed for data analysis. According to the analyzes, it was observed that 5 min jogging and 15 s stretching exercises increased the isokinetic strength, whereas 30 and 45 s stretching exercises caused a decrease.

## 1. Introduction

Static stretching at various durations has been shown to be a widespread practice among athletes during training sessions for different muscle groups or before competitions [1]. Static stretching can lead to an acute increase in the angle of motion [2]. However, recent research has shown that stretching applied to various muscle groups may have adverse effects on maximal muscle strength performance [1,3,4,5]. Although positive effects of the static stretching on the flexibility have been observed [6,7,8], there are studies concluding that stretching has either negative [9,10,11] or no effect [9,12,13,14] on maximal strength and explosive strength. As opposed to several studies conducted on sedentary in which strength performance was shown to decrease upon increased active static stretching duration, there are studies declaring that strength performance is not affected by stretching when the same protocol was applied to active athletes [10,11,15,16].

The effects of static stretching on different exercise performances of athletes have also been subject to much research. In a study conducted in 2004, no statistically meaningful change in jumping performance was found upon measurements made after static stretching (SS) [17]. In addition, several studies have found that static stretching has negative effects on the explosive strength and jumping performance of athletes [18,19,20,21].

As muscle strength is evaluated, Hamstring/Quadriceps (H/Q) muscle ratio is one of the most important parameters in determining the muscle balances of athletes [22]. A significant relationship between H and Q is important for the knee joint. In addition, determination of this ratio by isokinetic tests is necessary for the protection of muscle strength balance, thus to prevent injuries. Stretching exercise programs have been reported to be able to make positive contributions to muscle strength balance and function by providing motion coordination and strength development [23,24]. Peak torque H/Q ratio measurement for hamstring and quadriceps muscle groups is utilized to better understand the positive effects of rehabilitation therapies for injured athletes and improvement of athletes’ performance. In addition to its utility for determining the strength balance between the agonist and antagonist knee muscles and the function and stability of the knee joint, H/Q ratio is also used to investigate the strength and balance between hamstring and quadriceps during speed-dependent motions [25,26,27,28].

In recent years, stretching exercises have been an important discussion topic for coaches and athletes in terms of its impacts on performance of elite athletes. It is also stated that warm-up and stretching exercises are important parts of warming up exercises due to their possible effects on injuries and performance, while at the same time they are accepted as a means of preparing athletes’ musculoskeletal system for the exercise before any physical activity [24,25,27]. There are studies supporting the fact that stretching exercises using static, dynamic, ballistic or Proprioceptive Neuromuscular Facilitation (PNF) stretching techniques have positive effects on joint mobility. Increased mobility results in reduced muscle disability and consequently better sport performance [29,30,31,32]. However, there are also other studies revealing that there is no negative or significant effect of stretching on maximal strength and explosive strength [9,12,13,14]. However, investigations conducted in this area focus on various forms of static stretching with undeniable positive effects and minimized negative outcomes. These forms are shaped around diverse static stretching exercises, varying durations and different time intervals. To contribute to the studies and the information presented so far, the purpose of this study is to determine the duration-dependent acute effect of static stretching applied to elite athletes on isokinetic strength of hamstring and quadriceps muscles and to demonstrate the effect of different stretching durations on isokinetic strength, as well as the optimal stretching duration.

## 2. Materials and Methods

This study was carried out in Gazi University Sports Sciences Faculty. The voluntary participants were by elite male athletes who competing in the first league; 10 from football and five from basketball branches. The measurements were carried out in the Measurement and Evaluation laboratory at Gazi University, Faculty of Sport Sciences. Prior to the study, the subjects were given detailed information about the procedure and voluntary consent form was read and signed by all participants. Permission has been obtained from the ethics comity of Gazi University Faculty of Medicine to be able to conduct the research. A total of four different experimental protocols were applied to each subject with at least a one-day interval, and measurements were made at 60°/s and 180°/s angular velocities. These measurements were made on different days. Static stretching was intended for special muscles groups (quadriceps, hamstring, calf, adductor, hip rotator). Different experimental protocols are as follows:

In the first protocol, isokinetic right leg measurement was made immediately after low intensity aerobic jogging for 5 min.

In the second protocol, isokinetic right leg measurement was made right after 5 min low intensity aerobic jogging followed by static stretching (15 s × 17 different static stretching exercises) that affected hamstring and quadriceps muscle groups and 5 s rest was allowed between each exercise.

In the third protocol, isokinetic right leg measurement was made right after 5 min low intensity aerobic jogging followed by static stretching (30 s × 17 different static stretching exercises) that affected hamstring and quadriceps muscle groups and 5 s rest was allowed between each exercise.

In the fourth protocol, isokinetic right leg measurement was made right after 5 min low intensity aerobic jogging followed by static stretching (45 s × 17 different static stretching exercises) that affected hamstring and quadriceps muscle groups and 5 s rest was allowed between each exercises.

After each measurement, the isokinetic right leg hamstring, quadriceps, and hamstring/quadriceps strength ratios of all subjects were quantified by isokinetic dynamometer (Isomed 2000, D&R Ferstl GmbH, Hemau, Germany) as five repetitive measurements at 60°/s angular velocity (maximum strength) and 20 repetitive measurements at 180°/s angular velocity (explosive strength) [33]. Participants performed the protocol in the same order. For each measurement, five trials were made by the subjects prior to the test. During isokinetic strength measurements, subjects were supported by encouraging verbal assertions to improve their performance.

After the warm-up exercises, subjects participating in the study were immediately taken to the isokinetic strength measuring instrument one by one, and the dynamometer settings were made in accordance with the physical structures of each subject.

The test was performed in the sitting position. The subjects were fastened to the seat with the help of tapes from the middle of the abdomen and thighs. During the test, subjects were commanded to hold their arms on both sides of the seat to prevent the subjects’ arms from being released, and at the same time, this enabled additional support from the seat.

Statistical analysis of the data obtained in the study was performed using the SPSS 22 package program. Friedman variance analysis test can also be used in place of the F test for assumptions of normality and homogeneity in protocols. The Tukey HSD test was applied to reveal the differences between the variables. Statistical significance level was accepted as *p* < 0.05.

## 3. Result

The mean hamstring, quadriceps and ratio parameters obtained at 60° angular velocity for all 4 different experimental protocol measurements of right leg strength are represented in Table 1. Upon the analysis of the difference between the protocols, statistically significant (*p* < 0.05) difference was observed. Between the B–D protocols (between 15 and 45 s SS) were significantly different in hamstring muscle force. Between the B–D (between 15 and 45 s SS) protocols were significantly different quadriceps muscle force and between the A–D protocols (between 5 min jogging and 45 s SS) were significantly different in ratio. The mean hamstring, quadriceps and ratio parameters obtained at 180° angular velocity for all four different experimental protocol measurements of right leg strength are represented in Table 2. Upon the analysis of the difference between the protocols, statistically significant (*p* < 0.05) difference was observed. Between the A–C (between 5 min jogging and 30 s SS), A–D (between 5 min jogging and 45 s SS), and B–D (15 and 45 s SS) protocols were significantly different in hamstring muscle force and between the B–D (between 15 and 45 s SS) protocols were significantly different quadriceps muscle force.

## 4. Discussion

In this study, the duration-dependent effect of static stretching on quadriceps and hamstring muscle force was investigated. For the research group, four different experimental protocols were applied (A: only 5 min jogging; B: 5 min jogging + 15 s static stretching; C: 5 min jogging + 30 s static stretching; D: 5 min jogging + 45 s static stretching exercise). As the differences between the protocols were evaluated according to the results obtained in the study, significant differences were observed in the quadriceps and hamstring muscle group measurements at 60°/s angular velocity between B–D protocols after 15 and 45 s static stretching. Measurements at 180°/s angular velocity revealed statistically significant difference between A–C protocols (5 min jogging and 30 s SS), between A–D protocols (between 5 min jogging and 45 s SS) and finally between B–D protocols (between 15 and 45 s SS) for hamstring muscles. Significant differences were observed for the quadriceps muscle measurements at 180°/s angular velocity between B–D protocols (between 15 and 45 s SS). After the right leg strength measurements of the subjects for five times at 60° isokinetic angle following four different experimental protocols; when values of Hamstring and Quadriceps muscles are compared, it was observed that the best results were obtained after 15 s static stretching; while 5 min jogging, 30 s and 45 s stretching showed decrease in strength parameters.

After the right leg strength measurements of the subjects for 20 times at 180° isokinetic angles following four different experimental protocols; when hamstring peak torque values were compared to other stretching exercise results, it was observed that the differences were between in A–C and A–D protocols. When comparing the values of the quadriceps muscles, it was observed that the best result came after static stretching for 15 s whereas 5 min jogging, 30 and 45 s stretching exercises caused a decrease in the strength parameter.

There are many studies about the acute effect of static stretching exercises on strength, when made prior to the strength performance [18,19,20]. In these studies, static stretching exercises have often been found to have a negative acute effect on isokinetic strength performance. However, it can be difference according to the branches and different durations.

Research previously conducted by Zakas and colleagues on 15 professional sportsmen investigated how the effect of static stretching duration (1 × 30 s, 10 × 30 s, 16 × 30 s) on dominant leg knee extensor peak torque production varies at different angular velocities (60, 90, 150, 210 and 270) [34]. The research revealed that 1 × 30 s stretching protocol does not cause any change in torque production; however, 10 × 30 s and 16 × 30 s static stretching protocols decrease torque production at all indicated angular velocities and thus negatively affect isokinetic strength performance. In this research, the best result is in static stretching for 15 s, and Zakas did not use static stretching for 15 s. However, the same result was achieved in 30 s of static stretching.

Also, Zakas and colleagues, in separate study conducted on another 16 male footballers; investigated the effect of static stretching exercises on the isokinetic strength when applied at same angular velocity but for various repetition times. 15 s stretching for four times repetition and 15 s stretching for 32 times repetition were applied to the participants [19]. The study revealed that four times repetition protocol does not cause any change in isokinetic strength, whereas 32 times repetition protocol affects isokinetic strength negatively at all angular velocities.

In this research conducted, the static stretching durations and repetition times were reduced as compared to Zakas’s study; however, equivalent results were obtained.

Cramer and colleagues conducted a study on 13 women with an average activity of 1–5 h per week. In their study static stretching exercises were applied on dominant and non-dominant legs as four different motions (one motion performed without assistance, three motions with the help of an assistant), with four times repetition each for 30 s [15]. Their research, through the isokinetic tests at 60° and 180°/s angular velocity, revealed that the effect of static stretching on knee extensions isokinetic maximum torque production is not statistically significant. The same result was not obtained when we compared the study with this study. Cramer and colleagues have made four different movements in their study, but there is are different movements in this study.

In another study by Cramer and colleagues, the effect of stretching on knee extension isokinetic maximum torque production was analyzed on 14 sedentary women through static stretching applied as the same motion and for the same duration with the previous research. According to 60° and 240°/s isokinetic tests, the study revealed decrease in strength at both angle [10]. This study and the Cramer study came to the same conclusion.

As opposed to studies in which the strength performance is found to decrease as the static tension duration gets longer; there are a few studies showing that it is not affected.

Egan and colleagues conducted a study on 11 female basketball players in which 5, 15, 30, and 45 s of static stretching was applied on dominant leg lower extremity as four different motion (three assisted, one not assisted) and its effect on knee extensions isokinetic maximum torque production was analyzed at 60° and 300°/s angular velocities [11].

The research revealed that static stretching does not cause any change in torque production and there is not much of a strength loss as a result it does not coincide with this research. Egan and colleagues made four different movements in their study, which featured female basketball players.

When similar studies in the literature are examined, the results suggest that static stretching has a negative effect on strength performance. Researchers have often concluded that as the static stretching duration gets prolonged, it starts to have a negative effect on the isokinetic strength test at all angles. The discoveries of such research are parallel to the literature findings.

When the differences between the protocols were evaluated according to the obtained results, there was a significant difference between H/Q ratio of A–D protocols (after 5 min jogging and after 45 s SS) at the angular velocity of 60°/s. However, for the parameters obtained at 180°/s angular velocity, there was no significant difference in the H/Q ratio value between the groups. Zakas and colleagues found significant differences in H/Q ratio both at 60°/s and 180°/s angular velocities [2]. When compared to the study done, our study revealed the same result at 60°/s angular velocity, but it did not show parallel results at 180°/s. Zakas and colleagues conducted research on 15 elites and 13 amateur (in total 28) footballers and measured the peak torque, strength balance, and H/Q ratio at 12°, 60°, 180°, and 300°/s angular velocity by an isokinetic dynamometer. No significant difference was found in peak torque values, but there were significant differences in right and left legs’ flexion and extension strength balance between the groups.

In another study, Sangnier and colleagues, through isokinetic testing of soccer players, found that quadriceps and hamstring muscle groups differed in 50-degree flexion and extension and in resistance at 180 degrees [35]. It was stated that H/Q ratio below 0.6 may cause muscle injuries for footballers. They also recommended the use of isokinetic measurements periodically to monitor changes in muscle strength during these training sessions. In separate studies of Andrade Mdos, Escamilla and their colleagues, it is emphasized that the H/Q muscle ratio should be between 0.50 and 0.80 at different angular velocities [33,36]. In this study, the H/Q ratio is, as in the above literature, between 0.50 and 0.80.

As a result, in this study, it was observed that there is a loss of performance in isokinetic leg strength when the duration of stretching exercise is prolonged. As revealed by this research, static stretching practices may block hamstring and quadriceps muscle strength and thus affect the strength performance negatively. In the light of these results, it is suggested that static stretching exercises, especially above 15 s, should not be performed before strength trainings.

## Figures and Tables

**Table 1:** Hamstring, quadriceps, and ratio parameters obtained at 60° angular velocity.

Duration	N	Hamstring (N.M)	Quadriceps (N.M)	Ratio%	Difference between Protocols	*p* Value
5 min jogging (A)	15	202.087 ± 20.28	336.393 ± 37.07	59.67 ± 0.97	Ratio A–D	0.002
15 s SS (B)	15	216.413 ± 10.29	346.393 ± 12.81	62.67 ± 4.65	Hamstring B–D	0.041
					Quadriceps B–D	0.009
30 s SS (C)	15	210.540 ± 10.18	340.073 ± 7.37	61.67 ± 3.41		
45 s SS (D)	15	201.612 ± 16.85	314.240 ± 35.60	64.00 ± 2.23		

*p* < 0.05.

**Table 2:** Hamstring, quadriceps, and ratio parameters obtained at 180° angular velocity.

Duration	N	Hamstring (N.M)	Quadriceps (N.M)	Ratio%	Difference between Protocols	*p* Value
5 min jogging (A)	15	181.073 ± 9.74	303.560 ± 27.45	60.00 ± 6.91	Hamstring A–C	0.040
					Hamstring A–D	0.002
15 s SS (B)	15	179.647 ± 13.45	310.727 ± 19.84	58.00 ± 5.27	Hamstring B–D	0.005
					Quadriceps B–D	0.026
30 s SS (C)	15	166.407 ± 16.12	292.087 ± 15.21	56.67 ± 4.33		
45 s SS (D)	15	160.973 ±18.07	287.240 ± 23.99	55.67 ± 3.41		

*p* < 0.05.
